# Current Status of Tuberculosis and Human Immunodeficiency Virus Co-Infection in the Philippines: A Narrative Review

**DOI:** 10.3390/pathogens15101063

**Published:** 2026-10-08

**Authors:** Treena Rica D. Teh, John Joseph R. Chua, Scott Dean P. De Sagon, Mary Nicole I. Grecia, Michael Vencer Malaluan, Paul Benedic U. Salvador, Thea Coleen F. Sanico, Leslie Michelle M. Dalmacio

**Affiliations:** 1 College of Medicine, University of the Philippines Manila, Ermita, Manila 1000, Philippines; jrchua4@up.edu.ph (J.J.R.C.); spdesagon@up.edu.ph (S.D.P.D.S.); migrecia@up.edu.ph (M.N.I.G.); mvmalaluan@up.edu.ph (M.V.M.); pusalvador@up.edu.ph (P.B.U.S.); tfsanico@up.edu.ph (T.C.F.S.); 2 Department of Biochemistry and Molecular Biology, College of Medicine, University of the Philippines Manila, Ermita, Manila 1000, Philippines; lmdalmacio@up.edu.ph

**Keywords:** tuberculosis, human immunodeficiency virus, TB-HIV co-infection, Philippines

## Abstract

Tuberculosis (TB) and human immunodeficiency virus (HIV) co-infection remains a major public health concern globally and in the Philippines. The country continues to face a high burden of TB, multidrug- and rifampicin-resistant TB (MDR/RR-TB), HIV, and HIV-associated TB. Since HIV increases the risk of TB and TB worsens HIV outcomes, co-infection hinders progress toward the End TB Strategy and the UNAIDS 95-95-95 targets. This review summarizes the current knowledge on the epidemiology, prevention, and treatment of TB–HIV co-infection with a focus on the Philippine setting. Key challenges include socioeconomic barriers to care and rising drug resistance. In addition, the lack of an integrated TB–HIV data system disrupts continuity of care and weakens surveillance. Closing these gaps requires a coordinated national response that integrates effective policy with active community engagement. Research-driven and locally adapted strategies will be essential to bring the Philippines closer to its elimination goals.

## 1. Introduction

Tuberculosis (TB) is the leading cause of mortality from a single infectious agent worldwide [1]. Although it is highly preventable and curable, incidence rates have risen over the past few years following the COVID-19 pandemic, with an estimated 10.7 million new cases globally in 2024 [1]. Eight countries constitute two-thirds of these cases. Among these, the Philippines ranked third, accounting for 6.8% of cases [1]. Understanding the key determinants of TB infection and progression is therefore of paramount importance in addressing the disease burden and achieving the global End TB Strategy targets. These targets include reducing TB deaths by 90% and TB incidence by 80%, and eliminating catastrophic costs due to TB by 2030 [1]. Given the Philippines’ substantial contribution to the global TB burden, progress in the country will be essential to achieving these targets.

The strongest risk factor for TB is human immunodeficiency virus (HIV) infection, which affects 41 million people globally as of 2025, with 1.2 million new infections occurring annually [2]. HIV weakens the immune system and makes an individual 15–21 times more likely to acquire TB or develop recurrent TB [3,4,5]. Globally, in 2024, a total of 413,516 cases of TB were reported among people living with HIV (PLHIV) [1]. This constitutes 6.2% of the 6.7 million people that were newly diagnosed with TB and had an HIV-positive result [1]. This percentage of newly diagnosed TB individuals who had a positive HIV diagnosis has been declining in the past decade following a reported peak of 28% in 2006 [6]. In the same year, the Philippines reported an HIV-positive TB incidence of 4.7 per 100,000 population [6]. More recently, in the first quarter of 2026, 439 out of 109,967 new TB cases in the Philippines were reported as HIV-positive, 95% of which were new and relapse cases [7]. There is, however, no information on what proportion of these new TB cases were tested for HIV.

On the other hand, TB is the most common cause of morbidity and mortality among PLHIV, accounting for 40% of HIV-related deaths in resource limited settings [8,9]. Of the estimated 1.23 million deaths worldwide caused by TB in 2024, 150,000 were attributed to PLHIV [1]. Deaths from TB among PLHIV are officially classified as deaths caused by HIV/AIDS according to the International Classification of Diseases, with TB as a contributory cause [1]. This number has been in decline for several years and has fallen by 76% in 2024 compared to 2010 [1]. In contrast, recent national TB-HIV mortality data for the Philippines remains scarce, largely because local death certificates typically report immediate terminal complications (e.g., sepsis from disseminated TB) rather than the underlying co-infection [10]. Nevertheless, the TB-HIV syndemic remains a major global health concern, particularly in countries such as the Philippines, where rising HIV infection rates and socioeconomic challenges exacerbate TB outcomes in vulnerable populations [4,11,12]. These disparities underscore the urgency of strengthening both global and local efforts to combat the dual epidemic caused by TB-HIV co-infection.

In the Philippines, an estimated 1 million people have active TB, and surveillance reports from the first quarter of 2026 indicate a TB incidence rate of 95.71 per 100,000 people [7,13]. It is the 8th leading cause of death in the country, with about 70 Filipinos succumbing to the disease each day [14,15]. In addition, the World Health Organization (WHO) estimates the number of deaths due to TB in the Philippines to be 37,404 in 2024 [16]. Consequently, the Department of Health (DOH) launched the Philippine Strategic TB Elimination Plan (PhilSTEP), which aimed to reduce the TB burden in the country by decreasing the number of TB deaths to 5 per 100,000, decreasing the TB incidence to 300 per 100,000, and eliminating catastrophic costs incurred by TB-affected households by 2030 [17,18]. Meanwhile, the incidence of HIV infection in the Philippines is rapidly increasing, with a more than 400% increase in daily incidence over the past 10 years and an estimated 288,000 PLHIV in the country by the second quarter of 2026 [19,20]. Furthermore, the number of PLHIV in the country is projected to increase by more than 200%, from 158,400 in 2022 to 364,000 by 2030 [20]. The Philippines is committed to the 95-95-95 targets established by the Joint United Nations Programme on HIV and AIDS (UNAIDS), which aim for 95% of PLHIV to be aware of their status, 95% of diagnosed individuals to receive antiretroviral therapy (ART), and 95% of those on ART to have a suppressed viral load by 2030 [12]. Despite efforts to expand screening and treatment coverage, the country is falling behind in achieving milestones toward these goals, maintaining its status as a high-burden country for both TB and HIV-associated TB [1,16,21]. There is therefore a need to assess the present status of TB and HIV infection in the Philippines, evaluate the effectiveness of national programs, and identify existing gaps to accelerate progress toward more effective disease control and prevention.

Thus, in this review, we provide a comprehensive overview of the current knowledge of the epidemiology, pathophysiology, and risk factors for TB and HIV co-infection, with a particular focus on available data in the Philippine setting. We review the latest prevention and treatment strategies, as well as policies implemented to combat these diseases. We also highlight the challenges and barriers to achieving both global and local targets. Lastly, we identify critical gaps in the existing response and offer evidence-based recommendations to help mitigate the TB-HIV syndemic in the country.

## 2. TB and HIV Co-Infection

Human immunodeficiency virus infection remains a significant global health challenge. Among PLHIV, TB, caused by *Mycobacterium tuberculosis* (*Mtb*), remains the most common co-infection and the leading cause of mortality [13,22]. Co-infection with *Mtb* significantly accelerates the deterioration of immune function, particularly in high-burden regions such as the Philippines [13,22]. Understanding this synergistic interaction is critical to improving diagnostic accuracy, predicting disease outcomes, and identifying novel therapeutic strategies.

### 2.1. Immunopathogenesis of HIV Infection

Human immunodeficiency virus primarily targets CD4^+^ T cells, leading to progressive immunodeficiency and, ultimately, acquired immunodeficiency syndrome (AIDS) [23]. HIV enters the host through mucosal surfaces (particularly in the anogenital tract) where it is captured by dendritic cells (DCs) and presented to CD4^+^ T cells in lymphoid tissues [24,25]. The virus utilizes the CCR5 co-receptor to facilitate cellular entry and enhances DC maturation to promote viral dissemination [26].

The mechanisms of CD4^+^ T cell loss include apoptosis via caspase-3 in permissive cells and pyroptosis via caspase-1 in non-permissive cells—the latter of which results in systemic inflammation driven by IL-1β [27,28]. HIV also skews the immune response from a Th1 to a Th2 phenotype by reducing IFN-γ and IL-2 while promoting IL-4, IL-5, and IL-13 [29]. This ultimately impairs cell-mediated immunity [30]. Additionally, HIV-induced mitochondrial dysfunction and reactive oxygen species (ROS) production contribute to DNA damage, apoptosis, and chronic inflammation [31].

### 2.2. Immunopathogenesis of Mycobacterium tuberculosis (Mtb) Infection

Tuberculosis infection begins with inhalation of *Mtb*-containing droplets. The bacilli infect alveolar macrophages and modulate host cellular processes to evade destruction including prevention of phagosome-lysosome fusion and promotion of cytosolic escape [32,33]. *Mtb* induces the formation of granulomas, which are structured aggregates of immune cells that function to contain the infection. These granulomas consist of foamy macrophages, CD4^+^ and CD8^+^ T cells, dendritic cells, and multinucleated giant cells [34,35].

Despite being a host defense mechanism, granulomas can become sites of bacterial persistence. Their necrosis, often mediated by neutrophil infiltration, leads to bacillary dissemination through airways or hematogenous spread [36]. *Mtb* also suppresses MHC class II expression on DCs, dampening adaptive immunity [37].

### 2.3. Synergistic Mechanisms in Mtb-HIV Co-Infection

Human immunodeficiency virus significantly increases the risk of active TB even before CD4^+^ T cell counts fall below 500 cells/µL [38], suggesting that immune dysfunction precedes overt immunosuppression. This can be explained by the preferential depletion of *Mtb*-specific CD4^+^ T cells during early HIV infection, which undermines effective granuloma maintenance and TB control [39]. The loss of polyfunctional T cells producing IFN-γ, IL-2, TNF-α, and IL-17 exacerbates vulnerability to TB [40]. In addition, dysfunctional CD8^+^ T cells and impaired macrophage-mediated TNF-α apoptosis further compromise containment of *Mtb* [34,41]. Chronic HIV infection also leads to T cell exhaustion marked by PD-1, TIM-3, LAG-3, and CTLA-4 upregulation [42]. This is coupled with downregulation of co-stimulatory markers, such as CD27 and CD28, and expression of senescence markers, such as CD57 [42]. This results in an ineffective inflammatory state that leads to further progression of the disease. Conversely, active TB can worsen HIV outcomes. It accelerates CD4^+^ T cell depletion, increases systemic inflammation, and enhances viral replication via oxidative stress, glutathione depletion, and cytokine dysregulation [43,44,45]. *Mtb*-induced overproduction of TNF-α attracts HIV-susceptible cells, while T cell exhaustion promotes HIV spread via tunneling nanotubes [46]. Additional mechanisms include activation of latent HIV long terminal repeats (LTRs) and SOCS1 induction by *Mtb*, thereby facilitating viral replication and immune evasion [22,47].

Collectively, HIV and TB interact in a bidirectional and synergistic manner, resulting in an accelerated disease progression and worse clinical outcomes in individuals with co-infection. HIV-induced immunosuppression markedly increases the risk of developing active TB, while TB further enhances immune activation and accelerates HIV disease progression. This interaction complicates disease prevention, diagnosis, treatment, and long-term management, thereby causing a considerable strain on healthcare systems, especially in high-burden settings such as the Philippines. Understanding this interplay provides the foundation for examining the epidemiologic and contextual factors that drive HIV and TB co-infection in the Philippines. Furthermore, this understanding can inform a unified framework for improving health outcomes among individuals with TB-HIV co-infection.

## 3. Risk Factors for TB and HIV Infection in the Philippines

TB and HIV represent significant public health challenges, particularly in regions with high endemic rates of both diseases like the Philippines. Risk factors for TB-HIV co-infection are multifaceted and may involve host-related factors, microbial characteristics, health and behavioral factors, socioeconomic status, environmental factors, and public health efforts. Some risk factors may independently increase the risk of TB-HIV co-infection, as seen in immunocompromised individuals. However, other factors may be interrelated, resulting in a greater risk of infection. The co-occurrence of these risk factors has been observed in the Philippines and may contribute to the country’s morbidity and mortality. Currently, there are no local studies that compare these risk factors to determine the primary driver of the rise in TB-HIV co-infection in the Philippines. Nevertheless, these various risk factors likely influence the prevalence and transmission of TB-HIV co-infection by affecting the different epidemiologic factors of disease causation (i.e., agent, host, and environment). Hence, understanding the contribution of and interrelationship between these risk factors may prove useful in the development of strategies for prevention and intervention that are aimed at reducing the incidence of infection and improving patient outcomes.

### 3.1. Host Genetics

Communicable diseases such as TB and HIV infection may be influenced by host gene variants due to their effects on susceptibility, microbial clearance, and immune response and activation. Several candidate genes have been observed to alter the clinical progression of TB and HIV infection. These include various mutations and polymorphisms in genes involved in the innate immune response such as natural resistance-associated macrophage protein 1 (*NRAMP-1*), solute carrier protein 11A1 (*SLC11A1*), chemokines, cytokines, toll-like receptors (*TLR*), and human leukocyte antigen (*HLA*) [48,49,50,51]. Previous studies showed that mutations leading to dysfunctional NRAMP-1 lead to increased susceptibility to *Mtb* infection due to significant inhibition of intracellular killing of macrophages [49]. Furthermore, several polymorphisms in *NRAMP1* and *SLC11A1* have also been associated with increased susceptibility to TB infections across different populations [48,49]. Genetic variations in cytokines and chemokines (e.g., IFN-γ, IL-6, and IL-12) have also been implicated in mycobacterial infections. This is because polymorphisms in these genes can lead to impaired Th1 responses and macrophage activation [48,49,51]. These immune-related genes not only increase susceptibility to infection but may also predispose individuals to a more severe infection or to progression from latent to active TB [48].

Polymorphisms in immune-related genes such as TLRs, chemokines, cytokines, and HLA also have a similar effect on HIV infection, leading to higher viral loads and faster disease progression. However, findings on elite controllers of HIV, individuals who can suppress HIV without ART, have highlighted several notable genes that may be protective against HIV [52,53]. Mutations in genes involved in viral entry—such as chemokine receptors CCR5, CCR2, and CXCR4 and their respective ligands CCL3, CCL4, and CCL18—can significantly decrease susceptibility or hamper the progression of HIV infection [54]. For instance, individuals with homozygous loss-of-function alleles at the *CCR5* locus, which encodes the co-receptor of HIV gp120 for viral entry, showed resistance to HIV infection [55]. Increases in copy numbers and expression of ligands for CCR5 and CXCR4 have also been linked to protection against infection and disease progression. This is likely due to competitive inhibition through the physical blockade of virions from accessing the chemokine receptors [54]. Aside from these, genes related to immune response against viruses—such as killer cell immunoglobulin-like receptor (*KIR*), tripartite interaction motif 5 α (*TRIM5α*), and apolipoprotein B mRNA-editing enzyme catalytic polypeptide-like 3G (*APOBEC3G*)—are also involved in protective mechanisms against HIV infection by improving viral clearance and preventing or disrupting replication. Lastly, several studies found that polymorphisms in HLA genes such as HLA-B*57/58 and HLA-B*27 can lead to better prognosis marked by improved viral control and immune recovery [51,52,53]. All these findings highlight the importance of host genetics in determining the risk for TB and HIV infection.

### 3.2. Microbial Factors

Pathogenicity and virulence of *Mtb* and HIV may be affected by their lineages, subtypes, and variants. Genomic studies on *Mtb* isolates showed that the bacteria may be classified into nine lineages, with lineages 1–4 accounting for most of the clinical cases in endemic areas [56]. Some studies have found clinically relevant differences among lineages, particularly *Mtb* lineage 2 that is associated with higher prevalence of drug-resistance mutations against first-line treatment [57]. However, genetic sequence similarities among *Mtb* lineages are very high (reported at 94–99%) with almost no differences in pathogenic and virulence genes [56,58,59]. Furthermore, *Mtb* infection does not always lead to active disease [60]. Hence, despite evidence of variations in the distribution and virulence of *Mtb* lineages, the influence of *Mtb* genotype on the risk of infection remains to be fully elucidated [56,60].

In contrast, HIV types and subtypes have been associated with significant differences in disease burden and clinical outcomes. HIV-1 is the most prevalent type globally, while HIV-2 is less common, with its primary epicenter in West Africa [61,62]. HIV-1 has various subtypes associated with different geographic distributions and disease burdens. For instance, subtypes C and D have been linked with faster disease progression with greater viral load among PLHIV compared to other subtypes [62,63]. Several circulating recombinant forms (CRFs) of HIV-1 have also been identified in endemic areas including the Philippines [62]. Previous research showed that HIV-1 CRF01_AE is the predominant subtype in the country and has also been associated with higher viral loads and higher rates of resistance against tenofovir, which is one of the first-line antiretroviral drugs against HIV [64,65].

Another microbial factor contributing to disease severity is genetic variation. HIV rapidly generates diversity through spontaneous mutations, resulting in viral “quasispecies” that enhance viral replication, immune evasion, and drug resistance [66]. In the context of co-infection, these adaptive microbial traits impair host immunity, ultimately increasing the local community’s risk of acquiring, transmitting, and developing mild to severe TB and HIV clinical outcomes.

### 3.3. Behavioral Factors

High-risk behaviors—including but not limited to intravenous (IV) drug use, commercial sex, unprotected sex, and promiscuity—substantially elevate the risk of acquiring and transmitting HIV and other sexually transmitted infections (STIs) [2]. Moreover, these behaviors also reflect complex interpersonal exposure networks that increase susceptibility to other communicable diseases. For immunocompromised populations, such as individuals co-infected with TB and HIV, this heightened vulnerability extends beyond high-risk settings, where routine environmental and communal contact can similarly lead to severe opportunistic complications. Because behavioral factors greatly influence prevention and management, not just acquisition of infection, they are further discussed in Section 6.

### 3.4. Age and Sex

The latest data released by the DOH as of June 2026 reveal that individuals aged 25–34 years accounted for the largest proportion of new HIV cases (49%) followed by the 15–24 age group (30%) and the 35–49 age group (18%) [19]. Meanwhile for TB, as of March 2026, individuals aged ≥ 60 years comprised the largest proportion of new cases (28%), with case proportions declining across younger age groups [7]. With regard to sex distribution in HIV, a staggering difference can be observed as 94% of new cases were males as of June 2026 [19]. Similarly, among new TB cases as of March 2026, the majority (64%) of reported cases were males. In the same time period, TB-HIV co-infection was identified in 415 new and relapse TB cases, but no age and sex stratification were reported.

This lack of age- and sex-stratified national data limits the establishment of definitive epidemiological patterns of TB-HIV co-infection in the Philippines. However, a study on the social determinants of TB-HIV co-infection in Ethiopia show that ages 25–50 years accounted for most of TB-HIV cases (76.9%), followed by ages 15–25 years (13.9%) [67]. The higher number of cases in these age groups may be attributed to high-risk behavioral factors as previously discussed that may contribute to the risk of acquiring TB-HIV co-infection [68]. In the same study, TB-HIV co-infection was reported more frequently in females than in males (58.1%) and more than half of the mortality among respondents occurred in females (58.2%) [67]. The sex of patients may also affect the outcomes of TB-HIV infection due to behavioral differences. Studies have shown behavioral differences between sexes, such as lower medication adherence and poorer health-seeking behavior among males, resulting in higher prevalence and worse treatment outcomes for male TB and HIV patients [69,70].

Furthermore, while age-specific adjusted odds ratios (AORs) for mortality were reported in the Ethiopian cohort (AOR = 1.8, 95% CI: 0.6–5.8 for ages 35–44 years; AOR = 0.9, 95% CI: 0.3–2.8 for ages 25–34 years), these estimates cross the null value of 1 and lacked statistical significance [67]. These findings must then be interpreted with caution as mortality is multifactorial and may be confounded by the societal activities and habits associated with these age groups in specific cultural and geographical settings. This highlights the need to further explore these social determinants and demographic patterns in TB-HIV co-infection in the Philippines.

### 3.5. Malnutrition

In TB-HIV patients, malnutrition involves both macro- and micronutrient deficiencies from inadequate intake and aberrations in metabolic processes stemming from the infections themselves [71]. Malnutrition heightens the risk of morbidity and mortality, particularly from communicable diseases like pulmonary TB (PTB). The risk is even greater for those with existing comorbidities such as HIV infection [72]. Unmanaged HIV infection and AIDS cause and exacerbate malnutrition, consequently contributing to a worse immunocompromised state that further risks co-infection with PTB or other opportunistic infections [72]. Independently, an increased risk of developing an active TB infection in those with latent TB has been attributed to undernutrition due to the associated impaired immunologic responses like HIV immunosuppression [71]. TB and HIV infections are strongly correlated with each other, and both are individually linked with undernutrition as well [71]. This means a “triple burden” looms in patients with TB-HIV and malnutrition [71]. The 2021 Expanded National Nutrition Survey (ENNS) published by the Food and Nutrition Research Institute’s Department of Science and Technology revealed that chronic energy deficiency (CED) has a troubling prevalence of 7.2% in adults aged 20–59 years [73]. CED is described as a state of chronic underweight (body mass index < 18.5 kg/m^2^) that carries risks for impaired function and health, although the individual remains in an adapted energy balance [74]. This age group consistently comprised the majority of reported HIV cases, according to the DOH, in the same time period [21].

Collectively, these findings suggest that the identified age groups are not only vulnerable to HIV and TB infection but also to malnutrition, which increases their susceptibility to both diseases individually and to TB-HIV co-infection.

### 3.6. Socioeconomic and Environmental Factors

The 2021 ENNS also highlighted that the lower wealth quintiles had a higher prevalence of CED [73]. This accentuates the existing association between economic issues and vulnerability to disease. In a qualitative study of men who have sex with men (MSM) groups in Manila, respondents (especially those with low income) reported that testing centers were too far, had excessively long waiting times, and had operating hours that conflicted with their work schedules [72]. The last reason echoes the imposed socioeconomic burden of a supposedly simple screening, as evidenced by the reluctance to miss work. Interestingly, in some cases, belonging to a higher socioeconomic class also became a barrier but more as a product of stigma and “moral superiority” dilemmas expected in such a demographic [75].

Societal factors also push specific groups toward vulnerability, mainly through being overlooked as minorities and through non-inclusive health care support [20]. Vulnerable groups include the transgender population, persons deprived of liberty (PDLs), and people living with disabilities who have concerns and experiences that are often not represented in the general population [20]. Overseas Filipino workers (OFWs), who account for a notable proportion of HIV cases, also endure differences between health care systems abroad and locally [20]. A lack of focus on vulnerable groups can evidently lead to poor health outcomes, increased risk of comorbidities, and unmonitored cases, all of which can ultimately affect the broader population as well.

Despite the decrease in communal activities during the pandemic, PLHIV may still have been vulnerable to co-infection due to challenges in adherence and monitoring. Additionally, individuals living in shared or overcrowded households are at greater risk of acquiring PTB because prolonged and close contact with infected household members is a well-established driver of PTB transmission [76]. Similarly, cramped spaces for PDLs are expected to pose a problem for PTB transmission, which further endangers PLHIV in correctional facilities.

Stigma against PLHIV, HIV, and TB has been highlighted as drivers of increased risks for HIV, TB, and TB-HIV infections, and this interconnects with other risk factors [64]. This stigma stems not only from Filipino culture and society but also from misinformation or a lack of information [64]. For some, this manifests as misconceptions about symptoms, transmission, and moral standing, among others [72]. Poor health literacy affects both disease surveillance and treatment adherence, as evidenced in previous studies [64,72]. Although government authorities and influential organizations and public figures have intensified health literacy and advocacy efforts in recent years, some individuals at risk of or living with HIV and TB continue to avoid seeking information from trained personnel. Fear of stigma and judgment may discourage engagement with healthcare services, limiting access to accurate and reliable health information [72,77]. This is seen not just in the Philippines but in other countries as well [77,78,79].

Although the available evidence highlights several important risk factors, much of the existing data is derived from studies conducted in Manila and a limited number of regions, with some evidence originating from other countries. Expanding research across underrepresented regions of the Philippines is essential to develop a more comprehensive and representative understanding of the epidemiology of TB, HIV, and TB-HIV co-infection. Such investigation is critical for informing public health interventions that are aimed at reducing, and ultimately eliminating, the burden of these diseases in the Filipino population.

## 4. Strategies for Prevention of TB and HIV Infection in the Philippines

### 4.1. Reducing the Burden of TB Among People Living with HIV

Strategies to reduce the burden of TB among PLHIV include screening for TB, treating the diagnosed TB disease, and preventing TB among those who were negative for active TB during screening and evaluation. Treatment for HIV-associated TB is discussed in Section 5.

The Philippines adopts a policy of screening all PLHIV for TB co-infection upon diagnosis of HIV/AIDS, annually, and at every visit to a health center [79,80,81]. Upon diagnosis of HIV/AIDS and annually, TB screening is performed using the WHO-recommended four-symptom screen and chest X-ray. In contrast, at every visit, TB screening is performed using the four-symptom screen alone [80,81]. A patient is identified as having presumptive TB if they report the presence of any one of the four symptoms of cough, unexplained fever, unexplained weight loss, and night sweats of any duration and/or if they exhibit a chest X-ray suggestive of TB [80,81]. Further evaluation would then follow through the collection of a sputum sample for processing using the Xpert MTB/RIF assay [80]. Smear microscopy or TB loop-mediated isothermal amplification (LAMP) may be used as an alternative if Xpert is not available [80]. These strategies are consistent with the latest WHO guidelines [82]. Other recommendations from the WHO that are not yet included in the national guidelines include the use of C-reactive protein and the use of computer-aided detection software programs for interpreting digital X-rays for the screening of TB in PLHIV [82]. However, these are conditional recommendations with low certainty of evidence [82].

Prevention of TB among PLHIV includes administration of isoniazid preventive treatment (IPT) and infection control. According to the most recent national guidelines, all PLHIV aged 1 year and older are eligible to receive IPT [80]. Exclusion of active TB is done prior to initiation of IPT through TB symptom screening and chest X-ray for PLHIV aged 5 years and older [80]. In PLHIV aged 1–4 years old, screening of symptoms (e.g., poor weight gain, fever, current cough) and eliciting a history of contact with a person with TB is sufficient; a chest X-ray is not required prior to initiation of IPT in this age group [80]. Furthermore, testing for latent TB infection using the tuberculin skin test (TST) or interferon-gamma release assays (IGRAs) is also not required for eligible individuals [80,81]. Available IPT regimens in the country are similar to those recommended by the WHO, namely a 6-month course of daily isoniazid (6H), a 3-month course of weekly isoniazid and rifapentine (3HP), a 3-month course of daily isoniazid and rifampicin (3HR), or a 4-month course of daily rifampicin (4R) [80,83]. Individuals taking an isoniazid-containing regimen (6H, 3HP, or 3HR) are also prescribed pyridoxine (vitamin B6) 25 mg/day to prevent peripheral neuropathy [80,81].

The WHO-recommended guidelines for TB infection control among PLHIV are the same as those for TB infection control in the general population [82]. Similarly, local guidelines for TB infection control among HIV patients mirror those for broad TB infection control [79]. The latest Philippine clinical practice guidelines recommend adapting the 2019 WHO guidelines on TB infection control [83]. Briefly, these include administrative controls, such as the triage of people with signs and symptoms of TB or with TB disease; isolation of people with presumed or demonstrated TB infection; prompt initiation of TB treatment; and implementation of respiratory hygiene. These also include environmental controls, such as the use of upper-room germicidal ultraviolet (GUV) systems and ventilation systems, and respiratory protection, such as the use of N95 masks with consideration for cost and treatment setting [83].

### 4.2. Reducing the Burden of HIV Among People with Presumptive and Diagnosed TB

The strategies for reducing the burden of HIV among people with TB are broadly similar to those for reducing the burden of TB among PLHIV. These include screening for HIV, treating HIV upon diagnosis, and preventing HIV infection among presumptive and diagnosed TB patients.

The Philippines adopts a policy of routinely offering provider-initiated HIV counseling and testing to patients diagnosed with TB, which is consistent with the latest WHO guidelines [79,84]. While HIV testing is voluntary, patients who refuse HIV antibody testing will be offered counseling and testing during subsequent visits to the TB treatment facility [79]. Patients with a reactive result upon testing will be offered post-test counseling and referred to the national reference laboratory or a designated rapid HIV diagnostic algorithm (rHIVda) laboratory for confirmatory testing [79,84]. The WHO also recommends offering HIV testing services to household contacts, partners, and, in settings of high HIV burden, close contacts of patients with HIV-associated TB—although these specific recommendations are not yet incorporated into local guidelines [82]. However, there are local guidelines that stipulate for HIV testing services to be offered to social and sexual networks of PLHIV and members of the five key populations (i.e., MSM, sex workers, PDL and other enclosed settings, people who inject drugs, and trans and gender diverse people) [84,85].

Neither the WHO nor the Philippines has published recommendations, assessed using the GRADE methodology, on HIV prevention among presumptive and diagnosed TB patients [82]. However, general measures for prevention of HIV infection and transmission remain applicable. The WHO recommends a combination approach to HIV prevention whereby biomedical, behavioral, and structural interventions are all utilized [86].

One of the key biomedical interventions in HIV prevention and control is the use of ARTs as pre- and post-exposure prophylaxis (PrEP and PEP, respectively). Of the WHO-recommended PrEP products [87], only oral tenofovir (TDF) and emtricitabine (FTC) are included in the latest available national guidelines on PrEP in the Philippines [88]. However, other recommended PrEP products, such as oral tenofovir/lamivudine (TDF/3TC) and the dapivirine vaginal ring, are currently included in the national drug formulary [89,90]. Long-acting injectable cabotegravir, although not included in the formulary, has been approved by the Philippine Food and Drug Administration (FDA) [91]. With regard to PEP, for adults and adolescents, the recommended regimen of tenofovir, lamivudine (or emtricitabine), and dolutegravir (TLD) is FDA-approved and included in the national formulary [89,91,92]. However, of the alternative drugs to dolutegravir (DTG), only lopinavir with low-dose ritonavir (LPV/r) is FDA-approved and in the formulary [89,91]. Ritonavir alone and atazanavir alone are FDA-approved but not in the formulary, while darunavir (alone or in combination with low-dose ritonavir, DRV/r) and raltegravir have not yet been approved by the FDA [89,91]. For PEP in children aged 10 years and below, the preferred backbone regimen of abacavir and lamivudine (ABC/3TC) is included in the formulary [89,91,92]. However, its combination with DTG is not available [89,91]. Alternative dual-nucleoside reverse transcriptase inhibitor (NRTI) backbones, such as zidovudine/lamivudine (AZT/3TC) and tenofovir-based regimens (TDF/3TC or TDF/FTC), are also included in the national formulary. However, the co-formulation or combination of AZT/3TC with DTG remains unavailable in the public health supply [89,91].

## 5. Treatment and Prognosis of TB and HIV Co-Infection in the Philippines

Treatment and care for patients with TB-HIV co-infection necessitate a collaborative approach among various stakeholders [93,94]. This section discusses the current treatment approaches to TB and HIV in the Philippines, as well as the prognosis of patients with TB-HIV co-infection.

### 5.1. Current Treatment Approaches for TB and HIV Co-Infection in the Philippines

Vital to the effective management of TB-HIV co-infection are timely diagnosis, prompt initiation and proper timing of treatment, and monitoring and management of side effects while employing a patient-centered approach [93]. An integrated clinical practice guideline for these key aspects of TB-HIV management is still unavailable in the country. While current guidelines follow WHO recommendations [82], they are published separately and managed by two national programs under the DOH. These programs include the National Tuberculosis Control Program (NTP) and the National HIV, AIDS, and STI Prevention and Control Program (NASPCP). While there are efforts to integrate TB-HIV care delivery through administrative policies, such as Administrative Order (AO) No. 2022-0010 [95], its effective implementation remains to be seen across the country. The devolution of the country’s healthcare system is a significant barrier to the efficacy of national policies whose implementation depends on the decision-making and resources at the local government level [90]. Thus, the following subsections discuss the clinical management of TB among PLHIV and of HIV among patients with TB according to the 2021 and 2025 guidelines, respectively. The current status of implementation is also highlighted where local data is available.

#### 5.1.1. Clinical Management of TB Among PLHIV

Active TB among PLHIV is established through (1) bacteriological confirmation or (2) a clinical diagnosis [80]. Xpert MTB/RIF is the recommended primary diagnostic test for bacteriological confirmation of TB among PLHIV because it is more sensitive than smear microscopy and can detect resistance to rifampicin [82,83], which is the most effective first-line anti-TB medication. It can also be used on various specimen types such as sputum, cerebrospinal fluid, and blood, which is helpful for cases of extrapulmonary TB (EPTB) among PLHIV [82]. Alternatively, a clinical diagnosis can be made in the presence of signs and symptoms (e.g., cough and/or fever of any duration, weight loss, and night sweats lasting at least 3 weeks) and X-ray results suggestive of TB [81]. While Xpert MTB/RIF is the recommended diagnostic test, it only accounted for 74% of diagnosed cases in 2024, and the remainder were diagnosed clinically [80]. Nationwide, only 965 facilities are currently equipped with Xpert MTB/RIF diagnostic capacity, and scaling up this laboratory network remains an ongoing national priority [80].

Prompt initiation of anti-TB treatment among PLHIV has been shown to improve patient survival [82,96]. Current guidelines recommend starting empiric treatment among PLHIV with pending completion of diagnostic work-up if TB is highly suspected [81]. Guidelines for the medical management of active drug-susceptible TB disease among PLHIV do not differ from those for HIV-negative individuals. It entails selecting the appropriate treatment regimen, monitoring the treatment response, and monitoring and managing adverse events [80]. As of writing, the WHO and NTP recommend at least six months of daily directly observed treatment with 2HRZE/4HR: isoniazid (H), rifampicin (R), pyrazinamide (Z), and ethambutol (E) for the first two months (2HRZE, intensive phase), followed by four months of HR administration (4HR, continuation phase) [80,82,93]. The duration of treatment depends on the patient’s clinical presentation. PTB requires four months of the continuation phase, while EPTB involving the central nervous system, bones, or joints may require 10 months of HR therapy. Patients with delayed response to therapy also require 10 months of HR therapy [80,82,93]. The appropriate dosage of these medications is determined based on the patient’s weight and is provided in fixed-dose combinations to enhance compliance [80].

Proper timing of treatment initiation is critical in improving clinical outcomes, survival, and patients’ quality of life. It is also vital in preventing transmission of TB and HIV to their partners, household members, and community [93]. For ART-naive PLHIV with active TB, anti-TB treatment is initiated first [80] within 4 weeks of TB diagnosis [81]. This is followed as soon as possible by ART within 2 weeks of starting anti-TB treatment, regardless of CD4^+^ T cell count [82]. However, delayed initiation of ART, after the intensive phase of TB treatment, is recommended for PLHIV with TB meningitis [82]. This is to prevent TB immune reconstitution inflammatory syndrome (TB IRIS) [82]. PLHIV already on ART should be started on anti-TB treatment immediately and switched to an alternative first-line regimen [81] to reduce the risk of drug interactions (particularly with rifampicin) while maintaining virologic suppression. Similar to IPT, supplementation with pyridoxine 25 mg/day is also recommended for all PLHIV treated with isoniazid to prevent peripheral neuropathy [81].

#### 5.1.2. Clinical Management of HIV Infection Among TB Patients

Current local guidelines for the treatment of HIV infection among patients with TB are aligned with the United Nations (UN) Sustainable Development Goals’ (SDGs’) 95-95-95 targets and employ the “test early”, “treat early”, and “treat all” approach [96]. This approach was found to reduce the risk of mortality particularly among PLHIV with co-existing opportunistic infections such as TB [96].

Diagnosis of HIV infection among patients with TB is made in the presence of antibodies against HIV in a blood or tissue sample (i.e., seropositivity) [95]. Confirmatory testing uses WHO pre-qualified and FDA-registered HIV Rapid Diagnostic Tests (RDTs) following rHIVda by NASPCP [97]. However, official confirmatory testing can only be performed at a Certified rHIVda Confirmatory Laboratory (CrCL), creating an administrative burden [97]. Historically, most of the samples for confirmatory testing are sent to the National Reference Laboratory at the San Lazaro Hospital/STI’s AIDS Central Cooperative Laboratory in Manila, which often leads to delays in testing [97]. As of April 2026, 199 DOH-licensed CrCLs have been approved across the country, but only a third (142) of these are operational [98].

Standard ART regimens include a combination of two or three anti-HIV medications [99,100]. The choice of regimen is individualized based on the patient’s needs and characteristics. An effective regimen not only achieves virologic suppression, thereby prolonging the lives of PLHIV, but also reduces HIV transmission [99]. While a DTG-based regimen is preferred as first-line treatment for HIV infection [20,96], an alternative regimen (i.e., TDF + 3TC or FTC + efavirenz [EFV]) is preferred for PLHIV co-infected with TB who are taking rifampicin to prevent adverse drug interactions [96]. Treatment effectiveness and side effects among PLHIV on ART are monitored through regular clinical and laboratory assessments [96]. Ideally, viral load monitoring must be done within three to six months of ART initiation [96]. Determination of baseline CD4^+^ T cell counts for newly diagnosed PLHIV is also recommended when accessible [100].

#### 5.1.3. Importance of a Patient-Centered Approach in TB and HIV Care in the Philippines

A patient-centered approach is an integral principle of TB-HIV care worldwide [80,82,96]. In the country, service integration is legally mandated under Administrative Order No. 2022-0010 to uphold this principle [79,82,95]. However, significant work remains to be done to translate this policy into actual clinical practice [17,18,101]. Currently, the clinical management of TB among PLHIV and of HIV infection among TB patients remains separated within their respective designated treatment facilities [79]. The dependence on a fragmented referral system creates critical gaps in the continuum of care, leaving co-infected patients vulnerable to loss to follow-up [77].

To bridge these structural gaps and improve patient outcomes, provider-initiated HIV and TB counseling and testing, and administration of medication must be routinely employed in both service settings [79,80,93]. Active referrals by health workers based on likely HIV-related symptoms have been shown to link PLHIV to care in a timely manner [77]. Once enrolled, a patient-centered framework ensures the provision of comprehensive patient and family education, counseling, and psychosocial support [80,96]. Local studies validate that this approach directly promotes treatment adherence among PLHIV [20,77]. In particular, patient satisfaction with HIV and ART education provided by healthcare workers is strongly associated with good self-reported adherence and improved overall sexual and mental health [20,77]. Effective patient education also helps retain late-presenting PLHIV in long-term care [77]. Furthermore, integrating specialized care, such as gender-affirming services for transgender populations, shows potential for increasing their participation in the HIV care cascade [20]. This underscores the importance of support from health workers, community volunteers (e.g., *barangay* health workers), or family members in TB and HIV care in the country [80].

Since out-of-pocket health spending has been a major barrier to health-seeking behavior for many Filipinos, financial protection forms a critical component of patient-centered care. Under Republic Acts 10767 and 11166, standard anti-TB medications and ART are legally mandated to be provided free of charge by the government [102]. This is done through the inclusion of standard anti-TB medications and ART in the Philippine National Formulary [20,89] and the creation of insurance packages by the Philippine Health Insurance Corporation (PhilHealth) that cover medication, diagnostic tests, and consultations. Patients need to enroll in the respective TB and HIV treatment hubs designated by the DOH to access these packages: outpatient anti-TB Directly Observed Treatment Short-course (TB-DOTS) provides PHP 7800 (approximately USD 125) and Outpatient HIV/AIDS Treatment (OHAT) provides PHP 58,500 (approximately $935) in reimbursements annually as of 2026 [103,104,105]. While TB treatment hubs are mostly integrated into primary health care centers throughout the country, as of 2025, there are only 234 DOH-designated treatment hubs for free HIV services [103,104,105].

### 5.2. Prognosis of TB and HIV Co-Infection

Worldwide, TB is the leading cause of morbidity and mortality among PLHIV [82]. Individuals with TB-HIV co-infection have poorer treatment outcomes and increased mortality compared to their TB-positive, HIV-negative counterparts [94]. This is worsened by MDR-TB infections, which PLHIV are more susceptible to, making this specific patient population twice as likely to die during treatment [80,82,106,107]. This is consistent with the figures in a 2015 systematic review and meta-analysis of post-mortem studies of HIV-related death globally that reported TB to be the primary cause of death among PLHIV at 37.2% (95% CI: 25.7–48.7%), and that in almost half of these cases (45.8%, 95% CI: 32.6–59.1%), TB was undiagnosed before death [82]. Furthermore, in 2011, it was reported that the 10-year case fatality rate for untreated smear-positive and smear-negative TB is at 70% and 20%, respectively [107]. In the same study, it was reported that these numbers increase to 83% and 74% for the same categories among PLHIV [107]. Moreover, among patients with MDR-TB, HIV-positive individuals had the highest mortality rate, especially those not receiving ART [108].

Unfortunately, there are no local data on the prognosis and treatment outcomes of TB-HIV patients in the Philippines, but such information is needed to understand the burden of the disease in the country and inform policies, given that *Mtb* and HIV infection enhance each other, and both are significant public health problems at present [22,109].

## 6. Progress and Challenges of TB-HIV Care in the Philippines

While there are guidelines for both TB and HIV care in the Philippines and strategies to integrate them, several challenges still make implementation and attainment of targets difficult to achieve [102]. This section will go over the global and local targets and the progress and challenges in achieving these targets for TB and HIV. The section ends with a short discussion on how the progress and challenges for TB-HIV compare with those of either disease separately.

### 6.1. Global End TB Benchmarks and Philippine Strategic Plan (PhilSTEP) Implementation

Progress on TB control is measured against the targets set by the 2015 WHO End TB Strategy [1]. These targets include ending TB by 2035 through reducing TB incidence by 90% and deaths due to TB by 95% compared to the figures reported in 2015 [1]. Interim milestones set for 2025 include a 50% reduction in the TB incidence rate and a 75% decrease in the number of TB deaths, as well as the elimination of the catastrophic costs (equivalent to >20% of annual household income) of TB on affected households [1]. Toward these goals, global data show a net reduction of only 12% in the TB incidence rate between 2015 and 2025, and a net decrease of 29% in the number of TB deaths [1,16]. As of 2025, almost half of TB-affected households (47%) still experience catastrophic costs due to the disease, which include direct medical expenditures, nonmedical expenses, and indirect costs such as income losses [1].

In addition to the End TB Strategy, a UN high-level meeting on TB was held in 2023, which set nine targets for 2027. These are 90% TB treatment coverage; 90% TB preventive treatment coverage among high-risk populations; 100% coverage of rapid diagnostic testing for TB for new cases; 100% coverage of health and social benefits package for people with TB; initiation of the rollout of new, safe and effective TB vaccines within 5 years; provision of annual funding (22 billion USD) for universal access to quality prevention, diagnosis, treatment and care for TB; and annual funding (5 billion USD) for TB research [1]. Global progress towards these targets as of 2025 is as follows: 78% TB treatment coverage; 25% TB preventive treatment coverage among TB household contacts, and 58% among PLHIV; 54% coverage for rapid testing for TB; six TB vaccines in Phase III trials; 5.9 billion USD allocated for TB prevention, diagnostic and treatment services; and 1.2 billion dollars of funding secured for TB research [1]. In the same report, it was also noted that treatment success rates for drug-susceptible TB remain high at 88% (2023 data) and have improved for MDR/RR-TB at 71% (2022 data) [1].

Pursuant to these global targets, the Philippines developed PhilSTEP, which is currently in its second phase (PhilSTEP2), which spans the years 2025–2030 [18]. Under PhilSTEP2, the country aims to screen 10–13% of the population annually using chest X-ray; test 2–3% of the presumptive TB population; detect 90% of estimated incident TB cases; successfully treat 90% of diagnosed TB cases; and give TPT to 75% of eligible household contacts, in addition to the targets for reducing the TB burden [18]. By the end of PhilSTEP1, which spanned the years 2020 to 2023, the accomplishment of the set targets for TB prevention all fell below 50%. The number of individuals who were screened with chest X-ray reached 7 million of the targeted 50 million (14%). The number of individuals with presumptive TB who were tested reached 3.5 million of the targeted 12.8 million (27%). The number of individuals given TB preventive treatment (TPT) reached 108,735 out of the targeted 752,310 (14%) [18]. National data collected in 2017 report that 42% of TB-affected households suffer catastrophic costs due to the disease [1,16]. An updated estimate of this parameter is essential for understanding the effect of TB-associated costs on affected Filipino households [1]. However, no reports using more recent data are available at this time [1]. Other indicators for the provision of TB care in the country show 75% (95% UI: 46–140) treatment coverage, of which 74% were diagnosed using an RDT [1,16].

There has been a slight decrease in the incidence of MDR/RR-TB compared to 2015 figures, which currently stands at 27,869 (95% UI: 11,844–43,895), equivalent to a rate of 24 cases (95% UI: 10–38) per 100,000 population [1,16]. The Philippines belongs to the top 4 countries contributing to more than half of the global number of people estimated to have developed MDR/RR-TB in 2024—contributing 7.1% to the global incidence, ranking 3rd behind India (32%) and China (7.1%) [1]. More worrisome is that, in 2024, the Philippines was also among the top 5 countries accounting for the 60% gap between incident cases and those enrolled in treatment for MDR/RR-TB, at 9.3%, only behind India (33%) [1].

The current status of the Philippines in meeting the global and local End TB Strategy targets highlights the gaps that must be addressed through strengthened efforts in implementing TB control measures [1]. Though the increase in TB cases may be due to decreased COVID-related disruptions, an actual increase in the number of individuals acquiring the disease cannot be discounted and thus warrants attention. This emphasizes the need to evaluate the implementation of strategies for TB prevention (especially among vulnerable populations) and early diagnosis; administration of effective anti-TB treatment for drug-susceptible and MDR/RR-TB cases; execution of infection control programs; provision of adequate funding; and timely collection of national surveillance data to identify priority areas for intervention [1].

#### 6.1.1. Inadequate Government Funding and Lack of Social Safety Nets

One of the challenges faced by implementing programs is funding. Chest X-ray, which is crucial to TB screening, is an out-of-pocket expense and not covered by PhilHealth benefit packages [18,110], making this inaccessible to individuals of a lower socioeconomic status. Health service delivery has also been decentralized to local government units (LGUs). However, LGU funding for the implementation of these programs (such as procurement of anti-TB drugs) is limited [18]. Furthermore, there are disparities in each local unit’s capacity to screen and test individuals, resulting in variations in accessibility and quality of care [18]. Regarding TPT, although provided free of charge, it has not been prioritized by healthcare workers, resulting in low coverage [18]. There have also been reports of stockouts of TPT drugs and delays in delivery of TB medicines and commodities, resulting in interruptions of service delivery [110]. In addition, as stated previously, 42% of TB-affected families in the country continue to experience catastrophic costs stemming from non-medical expenses (e.g., transportation to healthcare centers) and income loss (e.g., due to time spent away from work or job loss) [1,16,94]. This is despite the availability of select free TB care services [94]. Moreover, these costs were significantly higher for those diagnosed with DR-TB and TB-HIV co-infection [94]. These reports underscore the importance of providing adequate social safety nets and assistance to TB patients.

#### 6.1.2. Antimicrobial Drug Resistance (AMR) Development

Antimicrobial drug resistance (AMR) is a formidable challenge in TB care in the country, particularly among PLHIV. A study using 2011 to 2018 data on the molecular epidemiology of *Mtb* infections among adult Filipinos with TB-HIV co-infection found that PLHIV have a 40% higher chance of getting infected with MDR-TB compared to their HIV-negative counterparts. Furthermore, the kind of infections they contract are commonly primary drug-resistant infections [111]. Treatment among PLHIV with MDR/RR-TB is also more challenging because of limited treatment options; several drug–drug interactions between the MDR-TB regimen and ART; and the high costs associated with these medications, especially over a longer duration (9 to 20 months) [80,111]. While careful consideration of medications is taken to avoid or to lessen adverse drug interactions and unfavorable side effects among patients co-infected with MDR-TB and HIV, this specific patient population still has poor treatment outcomes [80,111].

From the same study, 31.8% of *Mtb* isolates characterized were resistant to one of the first- or second-line anti-TB medications, including two MDR-TB isolates resistant to HRE and streptomycin [111]. This problem also extends to HIV where poor ART adherence can drive viral drug resistance [20]. In a study by Salvaña and colleagues (2020), 90.6% of PLHIV (*n* = 53) who experienced treatment failure after a year were reported to have drug-resistance mutations [112]. This study observed high rates of resistance to nucleoside/nucleotide reverse transcriptase inhibitors (NRTI), non-nucleoside reverse transcriptase inhibitor (NNRTI), and tenofovir (specifically due to K65R mutation). Evidence of multiclass resistance was also found [112]. These findings indicate a wider spectrum of resistance compared to a previous report that noted only highly resistant variants to NNRTIs (e.g., EFV and nevirapine [NVP]) in 2013 [113]. Transmitted drug resistance was also detected among 6.3% (*n* = 6) of treatment-naive PLHIV [114]. These findings not only threaten individual treatment outcomes but also broader population-level HIV management efforts [20].

One of the strategies to address the emerging problem of AMR is the use of drug-susceptibility testing to inform anti-TB medical management, and viral load testing and HIV drug resistance testing to inform HIV medical management [20,108]. Moreover, periodic genotypic surveillance of isolated *Mtb* and HIV strains is important for informing TB and ART treatment regimens in the country and expanding patient access to effective drugs especially for those with MDR-TB [108].

#### 6.1.3. Treatment Failure and TB Recurrence Among PLHIV

The risk of TB recurrence—defined as either a relapse (i.e., recurrence of a previous infection) or re-infection (i.e., a new infection with a different strain)—is higher among PLHIV, particularly those living in TB-endemic areas like the Philippines [115]. In a 2013 study on TB-HIV co-infection in the Asia-Pacific Region, it was reported that around 24.4% of PLHIV living in TB-endemic areas had TB recurrence within two years of successful anti-TB treatment. Importantly, the majority of these cases were re-infections [115]. It was estimated that the time to TB relapse was 8.1 months, while the time to TB re-infection was 20.1 months [115]. Additionally, MDR-TB was found to be a major contributory factor to TB treatment failure and true disease relapse [115].

Poor treatment adherence due to several factors, such as stigma, economic burden, lack of psychosocial support, and mental health disorders, have been identified as contributory to TB treatment failure among PLHIV [116]. Further discussion of these factors and how they affect the progress of TB-HIV care in the country is presented in Section 6.2.2.

Analogous to addressing the growing problem of AMR, there is a need for studies characterizing and monitoring TB re-infection, particularly among PLHIV, in order to support efforts that improve care from the individual patient level to population-level control programs [115].

### 6.2. Current Status and Hurdles to HIV Control and Prevention

Meanwhile, progress on HIV control is measured against the UNAIDS 95-95-95 targets. Progress made worldwide as of 2024 shows that 87% of PLHIV know their status, while 89% are receiving ART [12]. Of these individuals, 94% had achieved viral suppression [12]. The latest available surveillance data for the Philippines indicate that as of June 2026, 56% of the estimated number of PLHIV have been diagnosed, 69% of diagnosed PLHIV are on ART, and 57% of PLHIV on ART achieve viral suppression [7], figures that are below global targets. Additionally, the country aims to have 34,200 clients enrolled in PrEP services in 2024, 96,600 in 2025, and 153,200 in 2026 [117]. As of June 2026, a total of 107,567 individuals have enrolled in PrEP. The majority (78%) of these are from the National Capital Region or adjacent regions (Regions 3 and 4A) [7]. However, of the total enrolled individuals, only 25% returned for a PrEP refill in 2026 [7].

#### 6.2.1. Institutional and Financial Impediments to Achieving National HIV Strategic Targets

Failure to meet the global targets reveals gaps in the effective and adequate implementation of HIV strategies and services. Akin to TB programs, funding for HIV programs is limited, and LGUs vary in their capacities to provide accessible and adequate HIV services [101]. Training of private HIV healthcare providers is also not covered by the program, leading to a lack of manpower and limiting the avenues through which patients can access proper HIV services [110]. Despite the free medical services offered by the government, PLHIV without health insurance were found to have a significantly lower chance of initiating ART, and those who face the risk of losing their income discontinue HIV care after testing [20,77]. The associated cumbersome administrative processes and unclear procedures were also identified as barriers to the use of the OHAT package [102]. Additionally, oral PrEP, although available, is mainly supported by external funding sources [110]. Meanwhile, other PrEP products such as cabotegravir, which may be preferable due to its bimonthly dosing (compared to daily oral PrEP), is not yet available in the country despite being FDA-approved.

The COVID-19 pandemic also contributed to the shortcomings in achieving targets for HIV control and prevention. Disruptions to HIV service delivery during the COVID-19 pandemic in 2020 precipitated marked declines relative to 2019, including reductions in HIV testing (61%), new diagnoses (37%), ART enrollments (29%), and overall treatment coverage among estimated PLHIV (2%) [101]. Additionally, in 2020, prevention coverage among MSM and transgender women (TGW) decreased to 17% (from 26% in 2018), and condom use decreased to 36% (from 38% in 2018). Adherence challenges also emerged during the COVID-19 pandemic. In a cross-sectional study by Joves et al. (2023), respondents from different regions of the Philippines identified geographical and financial access to medication and treatment hubs as among the top barriers to treatment during this time [118].

However, even with the resumption of service delivery, progress still falls short of the targets. Viral load testing remains an area for improvement given its importance in guiding HIV prevention and control measures in the community, particularly among vulnerable groups [101]. Regardless of the pandemic, access to healthcare for HIV (and TB) has already been a challenge in the country. Disparities in medication supply were felt especially in the regions outside the capital [119,120]. Despite assurances from authorities that the national supply is stable, questions remain regarding the allocation and distribution of the medication across different regions of the country [120]. While the government has made efforts to steadily increase the capacity and number of treatment hubs [20], accessibility can still be challenging for other personal and economic reasons. Furthermore, the rise in HIV incidence was identified as the primary cause of the shortage [121]. Although HIV medications are provided free of charge, supplies are becoming increasingly limited, thus underscoring the need to scale up preventive measures.

Apart from the 95-95-95 targets, the country also aims for 95% of 10- to 24-year-olds in key populations to have basic knowledge of HIV transmission and prevention and to prevent new infections in this group as outlined in their 7th AIDS Medium Term Plan 2023–2028 [101]. However, in a 2021 survey, it was reported that only 1 in 5 young people has comprehensive knowledge of HIV. Additionally, 35% of young people did not believe that condom use reduces the risk of contracting HIV, and only a fifth of them who have ever had sex have used a condom [101]. This emphasizes the need for adequate information and education on HIV prevention [101].

Subsequently, good HIV treatment literacy was found to promote a higher chance of ART initiation [77]. PLHIV who were satisfied with how their health workers communicated information about HIV infection and ART were found to have good ART adherence. Interestingly, PLHIV with no formal education were found to be more likely to get HIV testing and care, which may be attributed to strong trust in their healthcare workers. In contrast, experiences of abuse and discrimination from healthcare workers, coupled with the fear of confidentiality breaches involving their healthcare records, impede initiation and retention in HIV care [77,102]. These findings reinforce the importance of doctor/healthcare worker-patient relationships in treatment initiation and healthcare, particularly in upholding medical confidentiality, and in reaching PLHIV and those with TB-HIV co-infection.

#### 6.2.2. Intersectionality of Stigma, Criminalization, and Systemic Barriers in HIV-Vulnerable Populations

Socio-cultural and economic factors, such as stigma, poor literacy, mental health and substance use disorders, are significant contributors to late HIV diagnosis and poor linkage to care. As such, these factors present substantial barriers to improved patient outcomes [20,77]. Stigma and discrimination towards PLHIV in the country stem from certain aspects of Filipino culture and society such as religious and fatalistic beliefs, machismo, criminalization of sex work/transactional sex, drug use, and abortion [20,102]. Notably, while TB has historically evoked significant stigma and discrimination due to fear of contagion, its magnitude remains secondary to the profound stigma associated with HIV [109]. Accordingly, those who have TB-HIV co-infection face greater stigma and discrimination relative to individuals with TB alone [109].

Stigma against HIV may lead to PLHIV purposefully avoiding detection and demonstrating poor health-seeking behaviors [122]. This exposes PLHIV and other vulnerable populations at risk of progressive immunosuppression that follows undetected and uncontrolled infection, increasing their susceptibility to opportunistic pathogens such as *Mtb* [122]. Among the most vulnerable to stigma and discrimination are young PLHIV (≤24 years old), female sex workers, MSM, transgender individuals, people living in rural areas, as well as OFWs [20,77]. Reports have stated that younger PLHIV, despite being more likely to get tested, have a significantly lower chance of initiating ART and poorer ART adherence due to stigma and discrimination, among other factors [20,77]. Female sex workers were also found to have a significantly lower chance of initiating ART and seeking HIV care [77]. Aside from stigma, the lack of healthcare benefits due to the nature of their work and their vulnerability to trafficking and exploitation are contributing factors to their poor health-seeking behavior [20]. The criminalization of drug use, transactional sex, and abortion have also hindered people engaging in, or who have engaged in, these activities from seeking proper care and advocating for their needs for fear of judgment and punishment [101]. People seeking or who have sought abortion require the same HIV services provided to pregnant people, as both circumstances can result from unprotected sex [101]. For people who use drugs and people who inject drugs, criminalization has also prevented the implementation of harm reduction programs, which are an important component of HIV prevention [101]. The recent “war on drugs” campaign by the government negatively affected HIV transmission and control, particularly among people who inject drugs, since the conditions resulting from this campaign induce poor practices such as sharing needles to avoid detection by authorities [102].

Persons deprived of liberty (PDLs) are also an important population at increased risk for HIV [20]. The prison environment and its management practices, poor quality of services, and the transitory nature of imprisonment all lead to poor initiation of and retention in HIV care [20]. Similarly, OFWs are vulnerable to poor HIV-related outcomes because of migration and working conditions abroad which hinder their ability to be linked to and retained in care [20]. People living in rural areas also face community stigma and experience poor access to care due to the considerable distance to social hygiene clinics and hospitals where ART and medical care are provided. Moreover, this cohort incurs out-of-pocket expenses to arrange transportation and accommodation in urban centers to access care [77,102].

Unfortunately, these barriers to care rarely exist in isolation, and PLHIV most often present with several risk factors at once. Thus, the majority of PLHIV in the country still seek medical care only when their condition is severe enough to require immediate medical attention (i.e., when they are already critically ill) [102].

### 6.3. Dual Burden Dynamics: Gaps in Systemic Integration, Pharmacotherapy, and Care Continuums in TB-HIV Co-Infection

In contrast to TB and HIV separately, TB-HIV co-infection does not have standardized global and local targets against which to measure progress. Nonetheless, successful treatment outcomes remain a challenge for PLHIV diagnosed with TB, with a success rate of 79% in 2023 [1]. Despite this, receiving both TB treatment and ART among patients with TB-HIV co-infection resulted in preventing 7 million deaths between 2010–2024 [1]. Global coverage of both HIV testing and ART administration among confirmed TB cases remains high at 82% and 91% in 2024, respectively [1].

#### 6.3.1. Systemic, Socio-Cultural, and Economic Barriers to Care

Fostering a patient-centered approach in TB-HIV care is a core strategy for attaining its targets and requires effective implementation [80,82]. Additionally, these barriers to care strengthen the need for an integrated multisectoral approach, with supporting legislation and evidence-based programs, to tackle TB-HIV treatment and control in the country [82]. Likewise, local integrated clinical practice guidelines tackling TB-HIV specifically are still unavailable. Although there is collaboration between the two national programs for TB and HIV, stronger leadership, governance, and coordination are still needed [95]. The referral system between the two programs and management pipelines should also be enhanced to ensure the continuity of care for co-infected patients [80,82].

Barriers to appropriate and effective TB-HIV care are largely the same as those of TB and HIV separately. Delivery of TB-HIV care suffers from the devolved healthcare system in the country. In addition, emerging antimicrobial resistance, pervading stigma, and out-of-pocket health expenditure are some of the issues that confront TB-HIV collaborative efforts, same as TB and HIV separately. Moreover, it is not unreasonable to think that the impact of these barriers on patient outcomes is further magnified in TB-HIV than in either disease alone.

#### 6.3.2. Drug–Drug Interactions Between Anti-TB and Antiretroviral Drugs

The clinical management of patients with TB-HIV co-infection remains challenging because of drug–drug interactions. These interactions decrease therapeutic efficacy and require dose adjustments that can affect the frequency and/or severity of side effects and adverse effects. Rifampicin, the most effective of the first-line anti-TB medications [1], reduces the concentration and exposure of DTG as well as of those of the alternative first-line drugs, rilpivirine (RPV) and AZT [123,124]. Thus, it is recommended that an additional 50 mg dose of DTG must be taken 12 h after the daily fixed-dose combination [20,123]. While addressing this may seem straightforward, the availability and procurement of a 50 mg single-formulation DTG tablet in the country is difficult, limiting the use of this drug in patients with TB-HIV co-infection [20,123]. In addition, the burden of taking multiple medications also puts TB-HIV co-infected patients at a higher risk of poor treatment adherence [116,125]. Table 1 presents a summary of these drug–drug interactions.

The rise of MDR-TB and antiretroviral drug resistance in the country limits treatment options, which is further complicated by drug–drug interactions, particularly among alternative and second-line regimens [111]. In the 2021 Philippine Clinical Practice Guidelines for the Diagnosis and Treatment of Adult TB, treatment recommendations for patients with TB-HIV co-infection include consideration of both rifampicin-based regimens (i.e., HRZE) and non-rifampicin-based regimens (i.e., moxifloxacin- and/or pretomanid-containing regimens) [111]. For PLHIV with MDR-TB on an EFV-containing regimen, replacement of EFV with NVP (if susceptible) is recommended since EFV decreases the concentration of bedaquiline (Bdq), which is a main component of the MDR-TB treatment regimen [80]. The concomitant use of Bdq by patients on a LPV/r-containing regimen should also be avoided because LPV/r increases Bdq concentrations threefold, thereby increasing the risk for adverse health outcomes [80]. Caution and monitoring are also warranted when linezolid, another MDR-TB drug, is taken with AZT as this combination can potentially cause bone marrow dysfunction, such as anemia [80].

Studies on the efficacy and safety of PLHIV with MDR-TB co-treatment remain scarce, representing an unaddressed gap in the literature [83]. More targeted trials in this patient group may be useful, as their complex clinical presentation often precludes their enrollment in larger trials among patients infected with TB or HIV alone. On the other hand, when they are enrolled, subgroup outcome analyses are not usually available [83,126]. As new evidence is generated from clinical trials (particularly those that evaluate the efficacy and safety of new drugs or treatment regimens for individuals with TB and/or HIV infection [126]), timely evaluation of their adaptability to the Philippine context would also help address drug–drug interactions and patient outcomes.

#### 6.3.3. Absence of Vaccines for TB and HIV

The lack of an effective vaccine for both TB and HIV remains an unmet need for the prevention of these diseases. Currently, the only TB preventive vaccine is the Bacillus Calmette-Guérin (BCG) vaccine, which is used in infants and has been suggested to decline in efficacy over time [127]. Thus, several TB vaccines are being investigated and developed for use in adolescents and adults, who are mainly age groups responsible for TB transmission [127]. Interestingly, the effectiveness of TB vaccine candidates in PLHIV is also being evaluated [128,129,130]. Several candidates have been shown to be immunogenic and safe in PLHIV; however, their efficacy against TB disease remains to be seen [128,129,130]. Further research is needed regarding the immune response to TB-HIV co-infection, as well as the timing and effects of ART and TPT on vaccine efficacy [128,129,130]. Nonetheless, it is worth mentioning that, in a previous study involving seven Asian countries, the Philippines was reported to be one of the two countries with the highest level of ART adherence, together with Bangladesh, suggesting that retention in care in the country is favorable when cases are diagnosed and engaged early [77,101]. Studies done abroad have shown that early linkage to care reduces all-cause mortality among PLHIV by 32%. Moreover, immediate ART initiation versus late initiation at CD4^+^ T cell count < 350 cells/microliter or at AIDS-defining illness results in a 53% decrease in risk of developing both severe AIDS and non-AIDS-related events (e.g., cancer, cardiovascular, renal, and liver diseases) [101]. Indeed, significant attrition persists in case uptake and retention across both the TB and HIV care cascades nationwide. Intensifying and continuing current efforts to engage and retain cases are imperative in achieving the WHO End TB Strategy and UN SDGs. This would be essential to stymie the surge in both TB (including MDR/RR-TB) and HIV cases in the country and to ensure improved quality of life among patients with TB-HIV co-infection.

## 7. Conclusions

TB-HIV co-infection is both a global and national health concern. HIV accelerates immune system deterioration, increasing the risk of active TB, while TB worsens HIV outcomes. Despite existing policies and strategies from government and non-government agencies, the Philippines remains a high-burden country for TB, MDR/RR-TB, and HIV-associated TB. Socioeconomic barriers, such as uneven access to screening and preventive services, poor health literacy and misconceptions, and late diagnosis with delayed linkage to care, pose persistent challenges in disease control. Emerging challenges, including rising MDR-TB, treatment failure, and HIV drug resistance, further complicate the care cascade. In addition, the absence of an integrated TB-HIV health record system disrupts continuity of care and weakens disease surveillance.

Addressing these barriers requires coordinated action across different levels of the government and the community. At the national level, the DOH should expand benefit packages to cover early viral load testing and essential laboratory monitoring. LGUs must mobilize health workers to deliver community-level support, particularly for vulnerable groups in geographically isolated and disadvantaged areas. Partnerships with NGOs are also vital in reducing stigma, providing psychosocial support, and ensuring inclusive delivery of services. Further research on preventive regimen uptake and completion, the effectiveness of new screening tools in the local setting, genomic surveillance of *Mtb* lineages and HIV subtypes, and recurrence of TB in PLHIV and vulnerable populations is also needed. Strengthening evidence in these areas will allow national policy to be more responsive to these emerging challenges. Finally, the implementation of an integrated TB-HIV registry would improve continuity of care, enabling stronger monitoring and evaluation, and tailored resource allocation.

**Figure 1:**
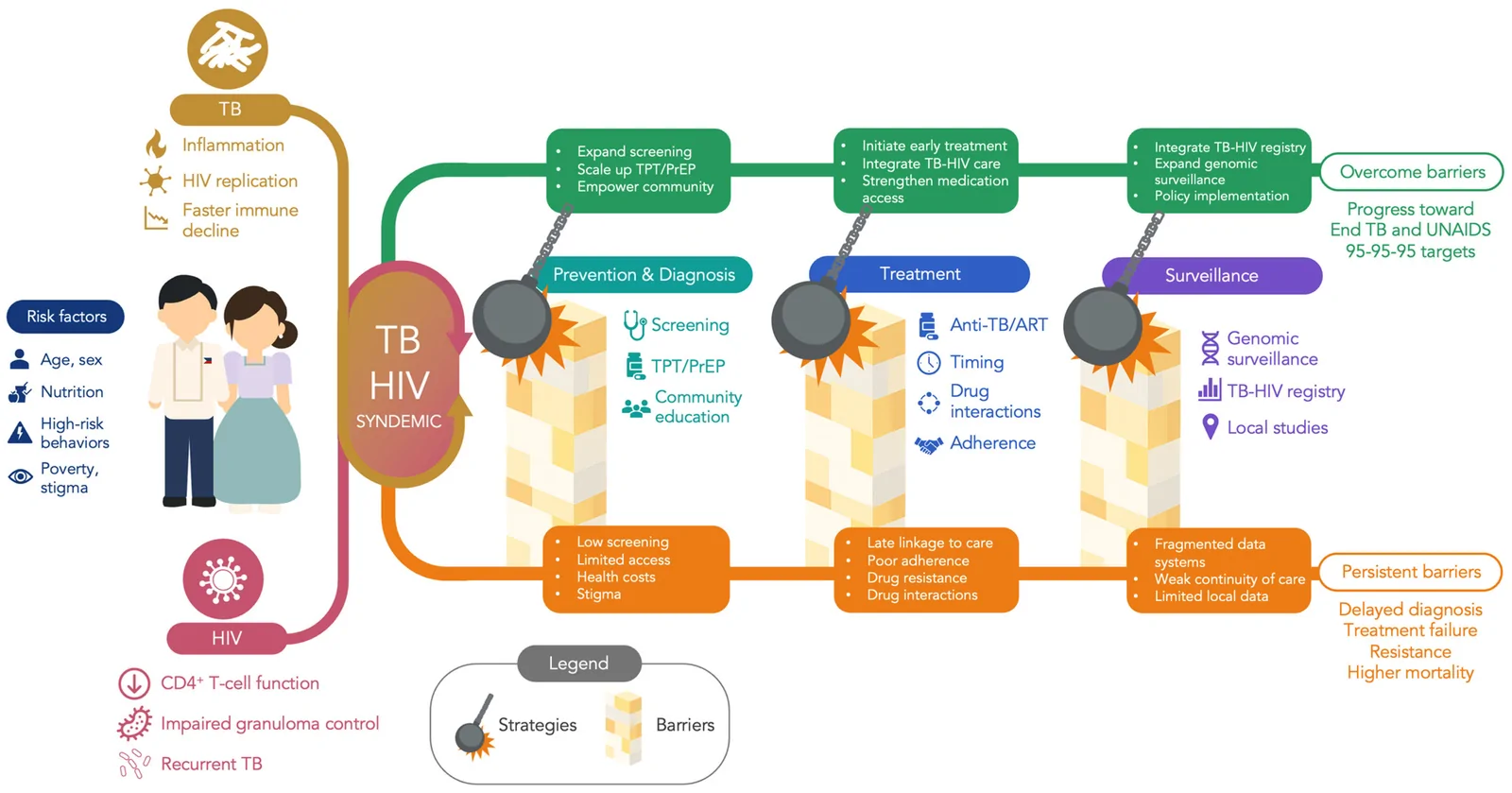
Overview of the TB-HIV syndemic in the Philippines. Tuberculosis (TB) and human deficiency virus (HIV) mutually exacerbate disease progression through interconnected effects. TB-associated effects are shown in gold and include inflammation, increased HIV replication, and accelerated immune decline. HIV-associated effects are shown in pink and include effects on T cell function and recurrent TB infection. These effects are further influenced by host and socioeconomic factors, including age, sex, high-risk behaviors, poverty, and stigma, which are shown in dark blue. Viewed from left to right, the TB-HIV syndemic is shown at the central left portion of the figure as the interaction between TB- and HIV-associated effects. Three key areas of intervention are as follows: prevention and diagnosis (teal), treatment (blue), and surveillance (purple). Pale-yellow brick walls represent barriers to these areas, with the specific barriers described in the dark-orange boxes. These include limited screening, stigma, poor adherence, drug interactions, fragmented data, and limited local data. The persistence of these barriers contributes to delayed diagnosis, inadequate treatment, treatment failure, drug resistance, and poor disease outcomes, ultimately contributing to increased morbidity and mortality. Gray demolition balls represent proposed strategies to overcome these barriers, with the corresponding strategies described in the green boxes. These include expanded screening and preventive therapy, early treatment, improved medication access and adherence, strengthened surveillance, and policy implementation. Together, these strategies aim to overcome the barriers to TB-HIV prevention, diagnosis, treatment, and surveillance, and may contribute to progress toward achievement of the WHO End TB Strategy and UNAIDS 95–95–95 targets in the Philippines. Abbreviations: TPT, tuberculosis preventive therapy; PrEP, pre-exposure prophylaxis; ART, antiretroviral therapy; TB-HIV, tuberculosis-human immunodeficiency virus.

A reinforced national response can move the Philippines closer to controlling TB and HIV co-infection and achieving its elimination targets. This requires coordinated policymaking, active community engagement, and research-driven, locally tailored strategies. As summarized in Figure 1, the TB-HIV syndemic in the Philippines is driven by the interplay of biological, socioeconomic, and health system factors. Progress towards elimination goals will therefore require integrated interventions spanning prevention, diagnosis, treatment, surveillance, and policy implementation.

## Figures and Tables

**Table 1:** Drug–drug interactions between the recommended anti-TB and antiretroviral drug regimens in the Philippines.

Anti-TB/Antiretroviral Drugs	Dolutegravir (DTG)	Abacavir (ABC) ^a^	Efavirenz (EFV) ^a^	Rilpivirine (RPV) ^a^	Zidovudine (AZT) ^a^
**Rifampicin**	**Dose-** **adjustment needed** Co-administration ↓ DTG concentration and exposure due to induction of the CYP3A and UGT1A1 enzymes by Rifampicin	No interaction	Potential weak interaction leads to a modest ↓ in EFV concentration but no recommended dose adjustment	**Contraindicated** Co-administration ↓ RPV concentration and exposure due to induction of CYP3A enzyme by Rifampicin, resulting in loss of virologic response	**Avoid coadministration** ↓ AZT concentration and exposure due to induction of the glucuronidation pathway by Rifampicin
**Bedaquiline** **(Bdq) ^b^**	No interaction	No interaction	**Avoid coadministration** Consider other ART drugs since EFV ↓ Bdq concentration through induction of the CYP3A enzyme	Potential weak interaction	No interaction
**Ethambutol**	No interaction	No interaction	No interaction	No interaction	No interaction
**Isoniazid**	No interaction	No interaction	No interaction	No interaction	No interaction
**Pyrazinamide**	No interaction	No interaction	No interaction	No interaction	No interaction

^a^ Part of the alternative first-line antiretroviral drug regimens in the Philippines. ^b^ Component of MDR/RR-TB drug regimen. Legend: ↓, decreases.

## Data Availability

No new data were created or analyzed in this study. Data sharing is not applicable to this article.

## Abbreviations

The following abbreviations are used in this manuscript:

2HRZE/4HR	Isoniazid (H), rifampicin (R), pyrazinamide (Z), and ethambutol (E) for the first two months (intensive phase), followed by four months administration of HR (continuation phase)
3HP	3-month course of weekly isoniazid and rifapentine
3HR	3-month course of daily isoniazid and rifampicin
3TC	lamivudine
4R	4-month course of daily rifampicin
6H	6-month course of daily isoniazid
ABC	abacavir
AIDS	acquired immunodeficiency syndrome
AMTP	AIDS Medium Term Plan
AO	administrative order
AOR	adjusted odds ratio
APOBEC3G	apolipoprotein B mRNA-editing enzyme, catalytic polypeptide-like 3G
ART	antiretroviral therapy
AZT	zidovudine
BCG	Bacillus Calmette-Guérin
Bdq	bedaquiline
CED	chronic energy deficiency
CrCL	Certified rHIVda Confirmatory Laboratory
CRF	circulating recombinant forms
DCs	dendritic cells
DRV/r	Darunavir with low-dose ritonavir
DOH	Department of Health (Philippines)
DOTS	Directly Observed Treatment Short Course
DTG	dolutegravir
EFV	efavirenz
EPTB	extrapulmonary tuberculosis
FDA	Food and Drug Administration
FTC	emtricitabine
GUV	germicidal ultraviolet
HIV	human immunodeficiency virus
HLA	human leukocyte antigen
IGRAs	interferon-gamma release assays
IPT	isoniazid preventive treatment
KIR	killer cell immunoglobulin-like receptor
LAMP	loop-mediated isothermal amplification
LGUs	local government units
LPV/r	lopinavir with low-dose ritonavir
MDR/RR-TB	multidrug- and rifampicin-resistant TB
MSM	men who have sex with men
*Mtb*	*Mycobacterium tuberculosis*
NASPCP	National STI and AIDS Prevention and Control Program
NGOs	non-governmental organizations
NNRTI	non-nucleoside reverse transcriptase inhibitor
NRAMP-1	natural resistance-associated macrophage protein 1
NRTI	nucleoside/nucleotide reverse transcriptase inhibitor
NTP	National Tuberculosis Control Program
NVP	nevirapine
OFWs	overseas Filipino workers
OHAT	Outpatient HIV/AIDS Treatment
OI	opportunistic infection
OR	odds ratio
PDLs	people deprived of liberty
PEP	post-exposure prophylaxis
PhilHealth	Philippine Health Insurance Corporation
PhilSTEP	Philippine Strategic TB Elimination Plan
PLHIV	people living with HIV
PMDT	Programmatic Management of Drug-resistant TB
PrEP	pre-exposure prophylaxis
PTB	pulmonary tuberculosis
RDTs	Rapid Diagnostic Tests
rHIVda	rapid HIV diagnostic algorithm
ROS	reactive oxygen species
RPV	rilpivirine
SDGs	Sustainable Development Goals
SLC11A1	solute carrier protein 11A1
TB	tuberculosis
TB IRIS	TB immune reconstitution inflammatory syndrome
TDF	tenofovir
TLD	tenofovir, lamivudine, and dolutegravir
TLRs	toll-like receptors
TPT	TB preventive treatment
TRIM5α	tripartite interaction motif 5 α
TST	tuberculin skin test
UI	uncertainty interval
UN	United Nations
UNAIDS	United Nations Programme on HIV and AIDS
WHO	World Health Organization
